# A Risk Prediction Model of Serious Adverse Events After Cardiac Catheterization for Chinese Adults Patients with Moderate and Severe Congenital Heart Disease

**DOI:** 10.31083/j.rcm2312415

**Published:** 2022-12-20

**Authors:** Juanzhou Hu, Yinghong Zhang, Wen Zhang, Jia Liu, Pan Peng

**Affiliations:** ^1^Medical College, Wuhan University of Science and Technology, 430065 Wuhan, Hubei, China; ^2^Department of Cardiology, Wuhan Asian Heart Hospital affiliated to Wuhan University of Science and Technology, 430022 Wuhan, Hubei, China

**Keywords:** adult congenital heart disease, catheterization, serious adverse events, risk prediction model, ROC curve

## Abstract

**Background:**

There are almost 2 million adult patients with congenital heart disease in China, and the number of moderate and severe patients is increasing. However, few studies have investigated the risk of serious adverse events (SAE) after catheterization among them. The aim of this study was to identify risk factors for SAE related to cardiac catheterization and to provide the risk scoring model for predicting SAE.

**Methods:**

A total of 690 patients with moderate and severe adult patients with congenital heart disease (ACHD) who underwent cardiac catheterization in Wuhan Asian Heart Hospital Affiliated to Wuhan University of Science and Technology from January 2018 to January 2022 were retrospectively collected and subsequently divided into a modeling group and a verification group. A univariate analysis was performed on the identified SAE risk factors, and then significant factors were included in the multivariate logistic regression model to screen for independent predictors of SAE. The receiver operating characteristic curve (ROC) and the Hosmer-Lemeshow test were used to evaluate the discrimination and calibration of the model, respectively.

**Results:**

A SAE occurred in 69 (10.0%) of the 690 catheterization procedures meeting inclusion criteria. The established SAE risk calculation formula was logit(*p*) = –6.134 + 0.992 × pulmonary artery hypertension (yes) + 1.459 × disease severity (severe) + 2.324 × procedure type (diagnostic and interventional) + 1.436 × cTnI (≥0.028 μg/L) + 1.537 × NT-proBNP (≥126.65 pg/mL). The total score of the final risk score model based on the effect size of each predictor was 0 to 7, involving pulmonary artery hypertension (1 point), disease severity (1 point), procedure type (2 points), cTnI (1 point) and NT-proBNP (2 points), and the score greater than 3 means high risk. The C-statistic of the area under the ROC curve was 0.840 and 0.911 for the derivation and validation cohorts, respectively. According to the Hosmer-Lemeshow test, the *p* values in the modeling group and the verification group were 0.064 and 0.868, respectively.

**Conclusions:**

The risk prediction model developed in this study has high discrimination and calibration, which can provide reference for clinical prediction and evaluation of SAE risk after cardiac catheterization in patients with moderate and severe ACHD.

## 1. Introduction

Congenital heart disease is defined as impaired formation of the heart and great vessels during embryonic period, or unclosed passage after birth, resulting in abnormal structure or function of the heart or great vessels [1]. With the advancement of pediatric cardiology, more and more children with congenital heart disease can successfully survive to adulthood [2]. There are almost 50 million adult patients with congenital heart disease (ACHD) worldwide [3], and the number in China increases rapidly, reaching about 2 million [4]. Cardiac catheterization has been widely applied to treat ACHD patients due to its advantages such as smaller incisions and fewer complications. However, studies have showed that the occurrence rate of serious adverse events (SAE) for ACHD patients underwent cardiac catheterization is as high as 24%, and compared with patients of mild ACHD, moderate and severe patients are more complex and have a higher chance of suffering from SAE after cardiac catheterization [5].

The risk prediction model can guide medical staff to carry out individual prevention and treatment measures [6]. Over the past 10 years several risk prediction models for ACHD patients after cardiac catheterization had been constructed [7, 8, 9]. However, there has been far less research conducted on SAE among Chinese patients with moderate and severe ACHD. Moreover, the application value of those models for Chinese patients has not been confirmed. The aim of this study is to develop and validate the SAE risk prediction model for Chinese moderate and severe ACHD patients, so as to help clinicians early identify the high-risk ACHD patients and provide timely prevention and treatment for them.

## 2. Materials and Methods

Our study was approved by the Medical Ethics Committee of Wuhan University of Science and Technology (reference number: 2022116). The data of all moderate and severe ACHD patients who underwent cardiac catheterization in Wuhan Asian Heart Hospital from January 2018 to January 2022 were retrospectively collected via the hospital electronic medical record system.

Inclusion Criteria: patients with congenital heart disease diagnosed by echocardiography; patients aged ≥18 years; disease severity in accordance with the “2020 ESC ACHD Guidelines” moderate and severe classification criteria [10] (**Supplementary Table 1**); patients undergoing cardiac catheterization; patients with complete case data. Exclusion criteria: patients with SAE occurred before the procedures; patients with related diseases that may lead to abnormal laboratory data (e.g., patients with preoperative myocardial infarction resulting in troponin elevation); patients with combined interventional and surgical procedures; cases with multiple cardiac catheterizations performed during a single hospitalization.

Among 690 patients included, 483 cases from January 2018 to December 2020 were used as the derivation cohort, while 207 cases from January 2021 to January 2022 were used as the validation cohort.

A SAE was defined as any adverse event causing mortality, permanent morbidity, need for further interventions, or extended length of stay [11] (**Supplementary Table 2**). SAE information recorded in the electronic medical record system included: event name, brief narrative description, identification time, symptoms, diagnostic auxiliary examinations, and handling measures.

There were 44 risk factors screened in our study: (1) General conditions: age, gender, height, weight, body mass index (BMI), heart rate (HR), systolic blood pressure (SBP), smoking or drinking history; hospital sources; (2) Complications: hypertension, diabetes, coronary heart disease, heart failure, cerebrovascular disease, chronic obstructive pulmonary disease (COPD), pulmonary artery hypertension, cyanosis, anemia; (3) Procedure-related indicators: disease severity, type of catheterization, procedure risk, access location, degree of surgical anesthesia, American Society of Anesthesiology (ASA) score. Procedure risk categories were devised based on the CRISP 9 and C3PO risk categories [12, 13] (**Supplementary Table 3**); (4) Laboratory examinations: N-terminal Pro-B-type Natriuretic Peptide (NT-proBNP), potassium determination, magnesium determination, calcium determination, cardiac troponin I (cTnI), uric acid (UA), triglyceride (TG), serum total cholesterol (TC), low-density lipoprotein cholesterol (LDL-C), high-density lipoprotein cholesterol (HDL-C), serum creatinine (Scr), serum urea (Urea), aspartate transferase (AST), lactate dehydrogenase (LDH), red blood cell (RBC), neutrophils (NEUT), hemoglobin (HB), high-sensitivity C-reactive protein (hs-CRP), and plasma D-dimer (D-D).

Data were analyzed using SPSS 26.0 software (IBM, Armonk, NY, USA). Data did not conform to normal distribution after inspected, so Mann-Whitney U test and Pearson chi-square test were used. Variables with statistical significance in univariate analysis were included in binary logistic regression analysis, and independent risk factors were screened by stepwise forward method for establishing risk prediction model. To eliminate the influence of extreme values on the regression results, continuous variables were dichotomously transformed using the cut-off value corresponding to the receiver operating characteristic (ROC) curve [14]. The discrimination of the risk prediction model was tested by the area under the ROC curve (AUC), and AUC >0.9 indicated high discriminatory power [15]. Hosmer-Lemeshow goodness of fit test was used to test the calibration, and *p *> 0.05 indicated good calibration [16].

Four methods were used to compare the application value between our model and CRISA model [7]: (1) –2log likelihood ratio (N2LL); (2) Akaike Information Criterion (AIC), defined as N2LL + (2 × k); (3) Bayesian Information Criterion (BIC), defined as N2LL + (ln (N) × k); (4) Area under the receiver operating curve (AUC).

## 3. Results

### 3.1 Baseline Characteristics

Among 690 moderate and severe ACHD patients who underwent cardiac catheterization, 236 (34.2%) were males and 454 (65.8%) were females, aged from 18–81 years with an average age of 44 years. The most common diagnosis was partial or complete atrioventricular septal defect (*n *= 226, 32.8%), followed by moderate and large patent ductus arteriosus (*n *= 86, 12.5%), aortic sinus aneurysm/fistula (*n *= 42, 6.1%), and congenital heart disease associated with pulmonary vascular disease (*n *= 37, 5.4%), as shown in Table 1. 529 (76.7%) procedures were diagnostic cardiac catheterization combined with interventional therapy and 161 (23.3%) procedures were isolated cardiac catheterization. Interventional procedures were most commonly closure (*n* = 371, 70.1%), followed by percutaneous transluminal angioplasty or stenting (*n* = 53, 10.0%), balloon valvuloplasty (*n* = 41, 7.8%), embolization (*n* = 38, 7.2%), and combined intervention for other complex malformations (*n* = 26, 4.9%).

**Table 1.** **Moderate and severe congenital heart disease classification and constituent ratio**.

Type of congenital heart disease		*n* (%)
Moderate		
	Anomalous pulmonary venous connection	18 (2.6)
	Anomalous coronary artery arising from the pulmonary artery	16 (2.3)
	Anomalous coronary artery arising from the opposite sinus	19 (2.8)
	Aortic stenosis — subvalvular or supravalvular	11 (1.6)
	Partial or complete atrioventricular septal defect	226 (32.8)
	Secondary atrial septal defect	25 (3.6)
	Coarctation of the aorta	16 (2.3)
	Double chambered right ventricle	22 (3.2)
	Unrepaired moderate and large patent ductus arteriosus	86 (12.5)
	Moderate or severe pulmonary stenosis	33 (4.8)
	Sinus of Valsalva aneurysm/fistula	42 (6.1)
	Sinus venosus defect	7 (1.0)
	Ventricular septal defect with associated anomalies	32 (4.6)
Severe		
	Congenital Heart Disease Associated with Pulmonary Vascular Disease	37 (5.4)
	Cyanotic congenital heart disease	26 (3.8)
	Double-outlet ventricle	21 (3.0)
	Interrupted aortic arch	3 (0.4)
	Pulmonary atresia	13 (1.9)
	Transposition of the great arteries	13 (1.9)
	Univentricular heart	12 (1.7)
	Truncus arteriosus	7 (1.0)
	Other complex atrioventricular conduction abnormalities and anomalous ventricular arterial connections	5 (0.7)

A total of 69 (10.0%) patients occurred postoperative SAE, of which 16 (2.3%) patients had two or more SAEs. The SAEs included arrhythmia requiring pharmacologic intervention (*n *= 23, 3.3%), pericardial effusion requiring surgical intervention or pericardial drainage (*n *= 12, 1.7%), pulmonary hemorrhag (*n *= 10, 1.4%), infection (*n *= 8, 1.2%), retroperitoneal hematoma (*n *= 8, 1.2%), arteriovenous fistula requiring surgical or transcatheter intervention (*n *= 6, 0.9%), secondary thoracotomy for hemostasis (*n *= 5, 0.7%), need for medicine or mechanical hemodynamic support (*n *= 4, 0.6%), unplanned transfusion (*n *= 4, 0.6%), anaphylactic reaction (*n *= 3, 0.4%), renal compromise (*n *= 3, 0.4%), pseudoaneurysm requiring surgical or transcatheter intervention (*n *= 3, 0.4%), complete heart block (*n *= 2, 0.3%), sudden cardiac arrest within 24 hours after operation (*n *= 2, 0.3%), death related to procedural complication (*n *= 2, 0.3%), hemothorax requiring thoracentesis (*n *= 1, 0.1%) and coronary artery thrombosis (*n *= 1, 0.1%).

### 3.2 Univariate Analysis

There were significant differences in 12 variables between SAE group and non-SAE group, including BMI, NT-proBNP, LDH, cTnI, Urea, NEUT, heart failure, pulmonary artery hypertension, severity of congenital heart disease, procedure type, ASA score, and access location (*p *< 0.05) (Table 2).

**Table 2.** **Baseline characteristics of derived cohorts**.

Variable		non-SAE (*n *= 432)	SAE (*n *= 51)	*p*-value
Age ${\mathrm{(years)}}^{a}$		43 (22)	44 (25)	0.310
${\mathrm{Gender}}^{a}$				0.164
	Male	152 (35.2)	23 (45.1)	
	Female	280 (64.8)	28 (54.9)	
Height ${\mathrm{(cm)}}^{b}$		1.60 (0.12)	1.61 (0.15)	0.120
Weight ${\mathrm{(kg)}}^{b}$		57.0 (13.7)	55.7 (19.0)	0.180
BMI (${\mathrm{kg/m}}^{2}$${)}^{b}$		22.4 (4.7)	21.0 (5.7)	0.010
HR (beats/${\mathrm{min)}}^{b}$		77 (14)	78 (22)	0.300
SP ${\mathrm{(mmHg)}}^{b}$		118 (19)	112 (27)	0.148
Hospital ${\mathrm{sources}}^{a}$				0.309
	Outpatient service	363 (84.0)	40 (78.4)	
	Emergency department	69 (16.0)	11 (21.6)	
Smoking ${\mathrm{history}}^{a}$				0.508
	Yes	27 (6.3)	2 (3.9)	
	No	405 (93.7)	49 (96.1)	
Alcohol ${\mathrm{history}}^{a}$				0.335
	Yes	27 (6.3)	5 (9.8)	
	No	405 (93.7)	46 (90.2)	
${\mathrm{Diabetes}}^{a}$				0.255
	Yes	15 (3.5)	4 (7.8)	
	No	417 (96.5)	47 (92.2)	
${\mathrm{Hypertension}}^{a}$				0.280
	Yes	46 (10.6)	8 (15.7)	
	No	386 (89.4)	43 (84.3)	
Coronary heart ${\mathrm{disease}}^{a}$				0.060
	Yes	59 (13.7)	12 (23.5)	
	No	373 (86.3)	39 (76.5)	
Heart ${\mathrm{failure}}^{a}$				0.012
	Yes	14 (3.2)	6 (11.8)	
	No	418 (96.8)	45(88.2)	
Cerebrovascular ${\mathrm{disease}}^{a}$				0.602
	Yes	20 (4.6)	1 (2.0)	
	No	412 (95.4)	50 (98.0)	
${\mathrm{COPD}}^{a}$				0.361
	Yes	3 (0.7)	1 (2.0)	
	No	429 (99.3)	50 (98.0)	
Pulmonary artery ${\mathrm{hypertension}}^{a}$				0.002
	Yes	150 (34.7)	29 (56.9)	
	No	282 (65.3)	22 (43.1)	
${\mathrm{Cyanosis}}^{a}$				0.056
	Yes	12 (2.8)	4 (7.8)	
	No	420 (97.2)	47 (92.2)	
${\mathrm{Anemia}}^{a}$				0.253
	Yes	9 (2.1)	3 (5.8)	
	No	423 (97.9)	49 (94.2)	
Disease ${\mathrm{Severity}}^{a}$				<0.001
	Moderate	343 (79.4)	28 (54.9)	
	Severe	89 (20.6)	23 (45.1)	
Procedure ${\mathrm{type}}^{a}$				0.019
	Diagnostic	94 (21.8)	4 (7.8)	
	Diagnostic and interventional	338 (78.2)	47 (92.2)	
Procedure risk ${\mathrm{category}}^{a}$				0.214
	Mild	248 (57.4)	26 (51.0)	
	Moderate	97 (22.5)	17 (33.3)	
	Severe	87 (20.1)	8 (15.7)	
Degree of ${\mathrm{anesthesia}}^{a}$				0.459
	Local anesthesia	406 (94.0)	46 (90.2)	
	General anesthesia	26 (6.0)	5 (9.8)	
ASA ${\mathrm{score}}^{a}$				<0.001
	1–2	382 (88.4)	30 (58.8)	
	3	35 (8.1)	5 (9.8)	
	4–5	15 (3.5)	16 (31.4)	
Access ${\mathrm{location}}^{a}$				0.014
	Arterial	15 (3.5)	6 (11.8)	
	Venous	283 (65.5)	36 (70.6)	
	Both	134 (31.0)	9 (17.6)	
Potassium ${\mathrm{(mmol/L)}}^{b}$		3.68 (0.34)	3.67 (0.53)	0.675
Magnesium ${\mathrm{(mmol/L)}}^{b}$		0.83 (0.08)	0.82 (0.07)	0.465
Calcium ${\mathrm{(mmol/L)}}^{b}$		2.31 (0.23)	2.28 (0.22)	0.194
NT-proBNP ${\mathrm{(pg/mL)}}^{b}$		54.22 (126.67)	242.9 (1044.78)	<0.001
cTnI ${\mathrm{(mg/L)}}^{b}$		0.004 (0.01)	0.006 (0.05)	0.007
AST ${\mathrm{(U/L)}}^{b}$		19.1 (7.3)	19.1 (12.3)	0.296
LDH ${\mathrm{(U/L)}}^{b}$		163 (47)	189 (70)	<0.001
Scr (μ${\mathrm{mol/L)}}^{b}$		66 (16)	69 (19)	0.098
Urea ${\mathrm{(mmol/L)}}^{b}$		4.96 (1.76)	5.40 (2.96)	0.041
UA ${\mathrm{(mmol/L)}}^{b}$		303 (117)	323 (144)	0.056
TG ${\mathrm{(mmol/L)}}^{b}$		1.21 (0.56)	1.21 (0.61)	0.420
TC ${\mathrm{(mmol/L)}}^{b}$		3.16 (1.66)	3.24 (1.50)	0.180
HDL-C ${\mathrm{(mmol/L)}}^{b}$		1.35 (0.45)	1.33 (0.32)	0.735
LDL-C ${\mathrm{(mmol/L)}}^{b}$		2.42 (0.58)	2.31 (0.99)	0.945
RBC (${\mathrm{10}}^{Ù}$${\mathrm{12/L)}}^{b}$		4.4 (0.7)	4.2 (0.5)	0.183
NEUT ${\mathrm{(\%)}}^{b}$		58 (14)	63 (17)	0.001
HB ${\mathrm{(g/L)}}^{b}$		132 (24)	133 (29)	0.881
Hs-CRP ${\mathrm{(mg/L)}}^{b}$		0.68 (1.12)	0.76 (1.9)	0.411
D-D ${\mathrm{(mg/L)}}^{b}$		0.21 (0.16)	0.22 (0.20)	0.523

Abbreviations: SAE, serious adverse event; BMI, body mass index; HR, heart rate; SBP, systolic blood pressure; COPD, chronic obstructive pulmonary disease; ASA, American Society of Anesthesiology; NT-proBNP, N-terminal pro-B-type natriuretic peptide; cTnI, cardiac troponin I; AST, aspartate transferase; LDH, lactate dehydrogenase; Scr, serum creatinine; Urea, serum urea; UA, uric acid; TG, triglycerides; TC, serum total cholesterol; LDL-C, low-density lipoprotein cholesterol; HDL-C, high-density lipoprotein cholesterol; RBC, red blood cells; NEUT, neutrophil; HB, hemoglobin; hs-CRP, high-sensitivity C-reactive protein; D-D, plasma D-dimer. ^a^N (%), *p* values from the Chi-squared test. ^b^M (IQR), *p* values from Mann-Whitney U test.

### 3.3 Development of the Risk Scoring Model for SAE

Multivariate analysis results showed that significant predictors of SAE included disease severity, procedure type, pulmonary artery hypertension, cTnI, and NT-proBNP (Table 3). The final established SAE risk calculation formula was logit(*p*) = –6.134 + 0.992 × pulmonary artery hypertension + 1.459 × disease severity (severe) + 2.324 × procedure type (diagnostic and interventional) + 1.436 × cTnI (≥0.028 μg/L) + 1.537 × NT-proBNP (≥126.65 pg/mL).

**Table 3.** **Multivariate analysis of risk factors for SAE in the derivation cohort**.

Variable	B	SE	Wald	*p*	OR	95% CI	
						Lower limit	Upper limit
Pulmonary artery hypertension	0.992	0.393	6.381	0.012	2.696	1.249	5.818
Disease Severity	1.459	0.375	15.130	<0.001	4.301	2.062	8.970
Procedure type	2.324	0.600	14.981	<0.001	10.217	3.149	33.148
cTnI	1.436	0.400	12.893	<0.001	4.205	1.920	9.208
NT-proBNP	1.537	0.382	16.226	<0.001	4.652	2.202	9.830
Constant	–6.134	0.725	71.533	<0.001	0.002	-	-

Abbreviations: B (Beta), Regression coefficient; SE, standard error; NT-proBNP, N-terminal pro-B-type natriuretic peptide; cTnI, cardiac troponin I; -, not applicable; CI, confidence interval; OR, odds ratio.

We converted the model into a scoring system. The weight of the predictor with the smallest βvalue is assigned to 1 point, and then the β value of other predictors is divided by the smallest βvalue, rounded to an integer to obtain the corresponding score (Table 4).

**Table 4.** **Risk prediction score of SAE**.

Variable		β	Points assigned
Pulmonary artery hypertension			
	No	0	0
	Yes	0.992	1
Disease Severity			
	Moderate	0	0
	Severe	1.459	1
Procedure type			
	Diagnostic	0	0
	Diagnostic and interventional	2.324	2
cTnI			
	<0.028 mg/L	0	0
	≥0.028 mg/L	1.436	1
NT-proBNP			
	<126.65 pg/mL	0	0
	≥126.65 pg/mL	1.537	2
Total score		-	0–7

Abbreviations: SAE, serious adverse event; β, Regression coefficient; cTnI, cardiac troponin I; NT-proBNP, N-terminal pro-B-type natriuretic peptide; -, not applicable.

### 3.4 Model Performance

**Fig. 1.**
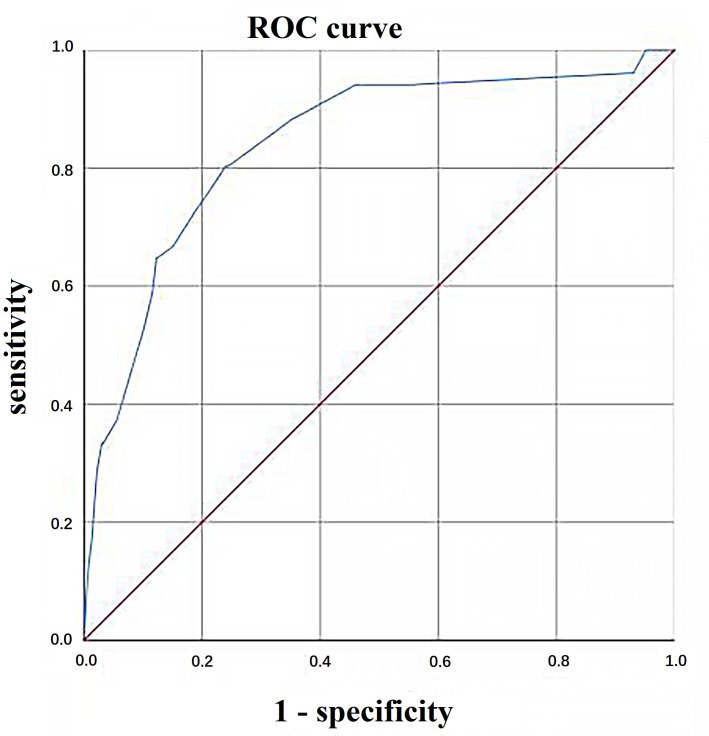
**Area under the ROC curve (AUC) plots for prediction model fitted on development sample**. ROC, receiver operating characteristic.

**Fig. 2.**
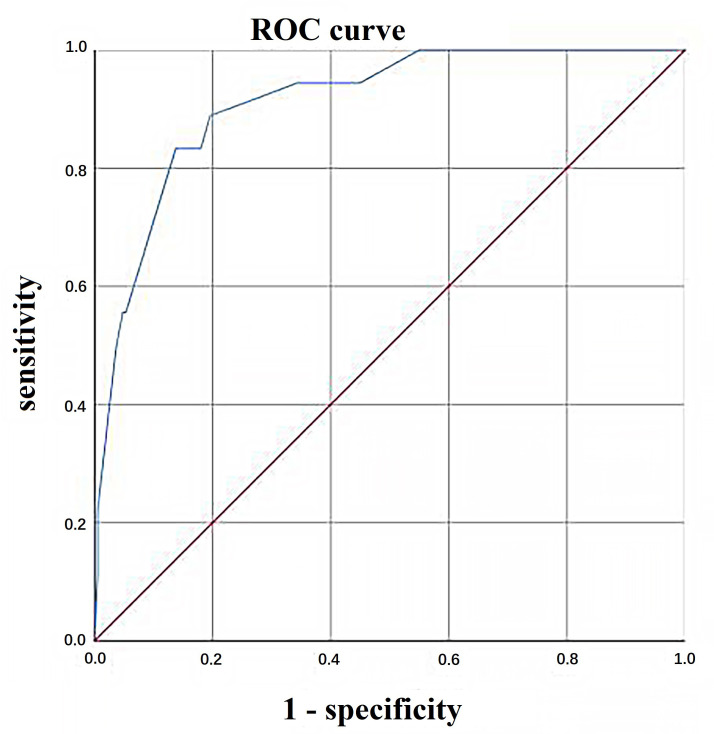
**Area under the ROC curve (AUC) plots for prediction model fitted on validation sample**. ROC, receiver operating characteristic.

**Fig. 3.**
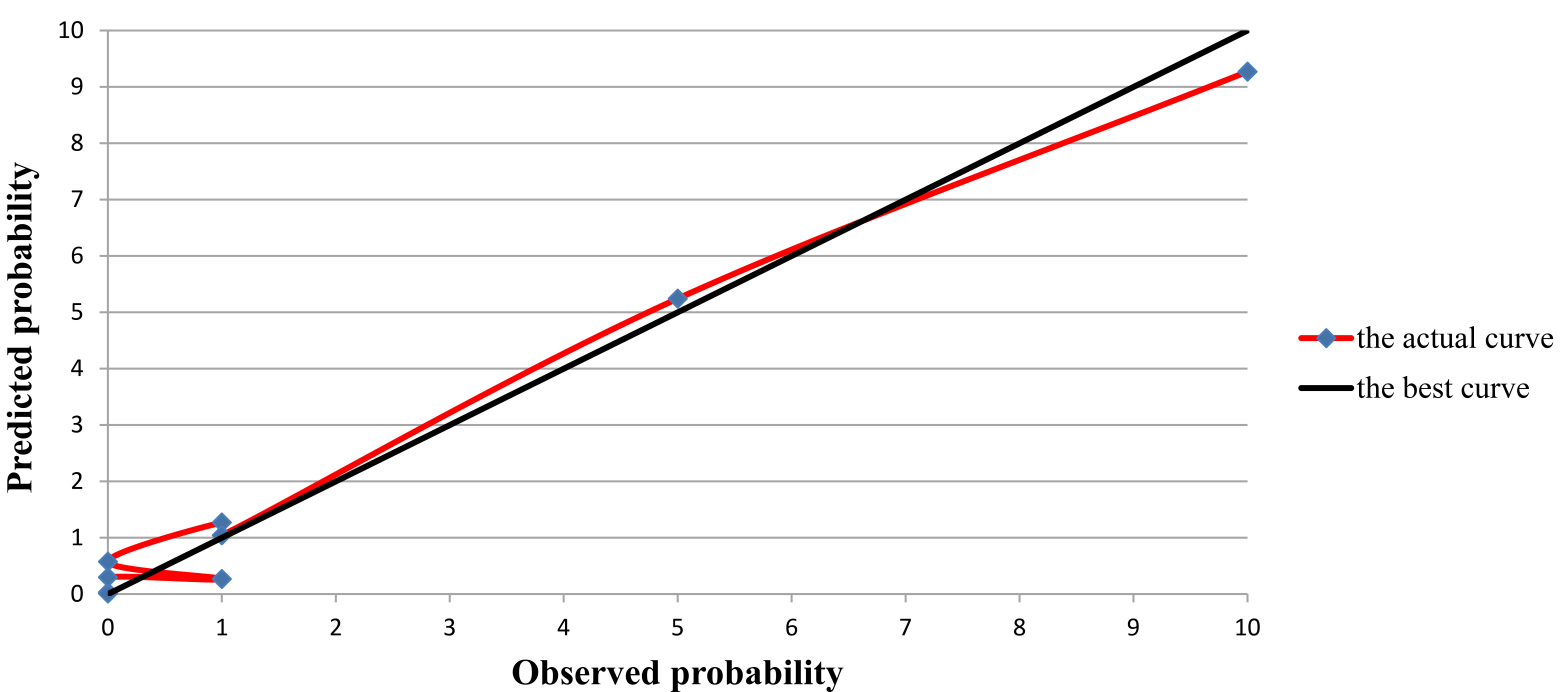
**Calibration curve for predicting the probability of SAE occurrence and the actual probability of SAE occurrence after cardiac catheterization in moderate and severe ACHD patients (The black line is the best curve and the red line is the actual curve)**.

The C-statistic for the incidence of SAE in the derivation and validation cohorts was 0.840 (95% CI, 0.779–0.901) and 0.911 (95% CI, 0.850–0.973), respectively, indicating that the model had a good degree of discrimination (shown in Fig. 1 and Fig. 2). In addition, the model showed good calibration, according to the *p* values of the Hosmer-Lemeshow goodness-of-fit test in the modeling group (χ^2^ = 10.414, *p* = 0.064) and the verification group (χ^2^ = 3.176, *p* = 0.868). Based on the calibration curve, there was a good agreement between the actual values and the predicted values in moderate and severe ACHD patients with SAE (shown in Fig. 3).

### 3.5 Application of the SAE Risk Prediction Model

To further simplify the SAE risk assessment model, the derivation cohort was divided into two groups: SAE low-risk group (0–3 points) and SAE high-risk group (4–7 points) according to the optimal cut-off value of 3.5 in the ROC curve of the scoring model [14]. In the derivation cohort, the incidence of SAE observed in patients in the low-risk and high-risk groups was 3.6% (13/358) and 30.4% (38/125), respectively, and the difference between the two groups was statistically significant (χ^2^ = 70.298, *p *< 0.001). In the validation cohort, the incidence of SAE observed in patients in the low-risk and high-risk group was 2.1% (3/141) and 22.7% (15/66), respectively, and the difference between the two groups was statistically significant (χ^2^ = 24.028, *p *< 0.001).

### 3.6 Comparison of Two Risk Prediction Models

Compared with CRISA model, we found that our risk prediction model had better application value for its lower N2LL, AIC, BIC, and higher AUC (Table 5).

**Table 5.** **Comparison of two risk score models**.

Risk prediction model	N2LL	AIC	BIC	AUC
CRISA model	92	–532	–502	0.777
Developed model	78	–563	–543	0.911

Abbreviations: CRISA, Catheterization RISk in Adult patients; AIC, Akaike’s Information Criteria; AUC, Area under the receiver operator curve; BIC, Schwarz’s Bayes Information Criteria; N2LL, –2log Likelihood (an assessment for model fit).

## 4. Discussion

Studies have shown that there are more than 1 million ACHD patients in the United States and Canada, and the number of moderate and severe ACHD patients increases rapidly [17]. Although the survival rate of ACHD is improved due to the development of cardiac catheterization techniques, serious complications and other unplanned adverse events after surgery are not rare, such as malignant arrhythmia and stroke, which will affect the patients’ clinical outcome, and sequentially increase the economic burden on them. In this study, we found a 10% incidence of SAE after catheterization in 690 patients with moderate and severe ACHD. In addition, we successfully developed and validated a risk prediction model for SAE and the model showed good discrimination (c statistic = 0.911) and calibration ability (χ^2^ = 3.176, *p* = 0.868). Finally, we transformed the model into a simple risk score and established risk stratification (a score greater than 3 means high risk) to provide catheterization risk assessment and consultation for patients with moderate and severe ACHD.

Up to now there has not been a SAE risk prediction tool for ACHD patients after cardiac catheterization in China. Many studies focused on the risk factors of ACHD and postoperative complications [18, 19, 20]. There were some risk prediction models developed in western countries.

Taggart NW *et al*. [7] developed a CRISA risk prediction model to predict the overall risk of SAE for ACHD patients with cardiac catheterizations, in which eight risk factors were included. Compared with this model, our model was more comprehensive, including not only above risk factors, but also other indicators such as past history and laboratory tests. Furthermore, it was worth noting that in CRISA model the types of procedure were classified into three categories: diagnostic, interventional and hybrid procedure. However, we found there was no separate interventional catheterization in China, because patients usually underwent intervention followed diagnostic procedure. Moreover, our research showed that our model was superior than the CRISA model in the comparison of model complexity and fit (Table 5).

Stefanescu Schmidt *et al*. [8] constructed a risk prediction model for major adverse events (MAE) after ACHD catheterization. Three features distinguished our model from their model. First of all, in the validation of the model, our model had a larger C-statistic (0.911 > 0.773) and better discrimination. Secondly, their study included adolescents over 10 years old, but our study targeted to ACHD patients over 18 years old. At last, besides events in MAE, other events such as bronchospasm were considered at the same time, so SAE was more extensive. In summary, our SAE included any complications that occurred after the procedure regardless of whether the underlying cause was catheterization or other aspects of the procedural care (e.g., anesthesia induction, airway management, etc.) [7].

Learn *et al*. [9] developed the model of congenital heart disease adjustment for risk method for adults with congenital heart disease (CHARM-ACHD), and claimed that adults underwent cardiac catheterization in pediatric hospitals in the past had fewer adverse events (4%). The model was included hemodynamic vulnerability indicators. However, in China, not every ACHD patient but severe patients are implemented comprehensive hemodynamic monitoring in ICU. Moreover, the application value of this model had not been validated in a separate cohort.

The SAE risk prediction model we constructed including three preoperative variables of pulmonary artery hypertension, NT-proBNP, cTnI, and two procedural variables of procedure type and disease severity. Pulmonary artery hypertension was a relatively common complication of congenital heart disease and associated with the size and nature of cardiac defects as well as environmental and genetic factors, accounting for approximately 10% of adult cases [21]. Compared with ACHD patients without pulmonary artery hypertension, pulmonary artery hypertension patients had a 2-fold increase in all-cause mortality and a 3-fold increase in the incidence of heart failure and arrhythmia, which somewhat increased the difficulty of catheterization procedures, such patients had a greater risk of postoperative SAE, and even experienced clinical deterioration after catheterization [22]. Type of procedure significantly predicted SAE, and our study found that the risk of SAE following diagnostic and interventional procedure was 2.324 times higher than diagnostic catheterization alone. At present, interventional therapy is widely used for ACHD patients to close intracardiac shunts, relieve obstructive valvular disease, stent stenotic vessels, replace and repair dysfunctional valves [23]. Compared with diagnostic catheterization, it will cause greater physical damage and a higher risk of postoperative complications.

Our result showed that patients with severe congenital heart disease had 1.459 times higher risk of SAE than moderate patients, which suggested that severity of ACHD was an important risk factor. In addition, our model included two biomarkers: NT-proBNP and cTnI. Preoperative NT-proBNP levels can be used as a marker to evaluate the hemodynamic and functional status of patients. Gessler P *et al*. [24] proposed that higher NT-proBNP was associated with ventricular dysfunction and ventricular volume overload in patients with ACHD. cTnI was a specific and sensitive marker [25], and Immer FF *et al*. [26] found that the maximum cTnI value within the first 24 hours of cardiac surgery can predict the serious postoperative complications, as well as the duration of intensive care treatment.

In our study, we confirmed that our model had good discrimination and calibration, and established simple risk stratification aimed at providing personalized risk counseling to patients before cardiac catheterization. Although the risks of catheterization may vary by random events, hospitals or surgeons, the risk score had a strong risk prediction power. Due to the intuitive and quantitative advantages of risk stratification, medical staff can use it to assess patients at high risk of SAE, so that personalized treatment can be adopted accordingly.

There were some limitations in this study. Firstly, the model included the overall SAE risk of moderate and severe ACHD patients after cardiac catheterization, lacking of the ability to predict risk of a specific kind of SAE. Secondly, the sample was limited to one hospital, therefore the model needs to be further validated in multiple center patients. Thirdly, we did not assess SAE risk of discharged patients but mainly focused on those of inpatients. Consequently, some potential factors may be omitted for the sake of focus.

## 5. Conclusions

A total of 690 moderate and severe ACHD patients who underwent cardiac catheterization were analyzed to identify procedural risk factors. We provided a prediction model for the risk of SAE after cardiac catheterization with favorable discrimination and calibration. The risk score scale developed based on the model could predict high-risk patients and allow medical providers to implement individualized prevention when cardiac catheterization is performed in ACHD patients.

## Data Availability

The data that support the findings of this study are available from [Wuhan Asian Heart Hospital] but restrictions apply to the availability of these data, which were used under license for the current study, and so are not publicly available. Data are however available from the authors upon reasonable request and with permission of [Wuhan Asian Heart Hospital].

## Supplementary Material

Supplementary material associated with this article can be found, in the online version, at https://doi.org/10.31083/j.rcm2312415
