# Supplementary material for: A Risk Prediction Model of Serious Adverse Events After Cardiac Catheterization for Chinese Adults Patients with Moderate and Severe Congenital Heart Disease
Source: Rev Cardiovasc Med. 2022 Dec 20;23(12):415. doi: 10.31083/j.rcm2312415 (PMC11270449; doi:10.31083/j.rcm2312415)
Supplement: Supplementary file 1 [file 2153-8174-23-12-415-s1.docx]

Supplementary Table 1: Qualifying Significant Adverse Events

| Access site re-bleeding requiring transfusion |
| --- |
| Air embolism requiring further treatment including hyperbaric therapy |
| Airway compromise, unanticipated. (Failed extubation, accidental extubation during procedure, need of intubation during / after the procedure, post-extubation airway obstruction requiring observation / intervention, extended need of mechanical ventilation |
| Anaphylactic reaction |
| Arrhythmia, hemodynamically unstable, requiring pharmacologic intervention |
| Arterial compromise requiring surgical or transcatheter intervention |
| Arteriovenous fistula requiring surgical or transcatheter intervention |
| Atrioventricular valve regurgitation significantly increased and related to the procedure |
| Balloon rupture causing vascular damage (requiring surgical or transcatheter intervention) |
| Bleeding / hematoma at access site requiring re-visit |
| Brachial plexus injury |
| Cardiac arrest/bronchospasm with induction |
| Cardiac arrest, unexpected, within 24 hours of procedure |
| Cardiac perforation |
| Complete heart block that is unresolved at the end of procedure |
| Device migration requiring open surgical removal, removal via cut down or transcatheter retrieval |
| Device migration, post procedure |
| Hemopericardium |
| Hematoma resulting in extended length of stay |
| Hemothorax |
| Infection (systemic/endocarditis), death related to procedural complication |
| Myocardial infarction |
| Pericardial effusion requiring surgical intervention or pericardial drainage |
| Pseudoaneurysm requiring surgical or transcatheter intervention |
| Pulmonary compromise (pulmonary hemorrhage / hemoptysis or pulmonary edema |
| Pulmonary embolus requiring intervention |
| Renal compromise |
| Retained device, unintentional |
| Retroperitoneal hematoma |
| Second organ system injury requiring extended hospitalization or therapeutic intervention |
| Seizure (new) within 48 hour of procedure |
| Separate procedure required emergently/urgently |
| Stent malposition (surgical or transcatheter intervention) |
| Stroke |
| Systemic embolism |
| Transfusion, unanticipated |
| Unanticipated escalation of hemodynamic support, CPS/mechanical circulatory support |
| Vascular injury (i.e. dissection, intimal tear, aneurysm) requiring surgical or transcatheter intervention |
| Venous compromise requiring surgical or transcatheter intervention |
| Wire embolization / retention requiring transcatheter retrieval or removal via cut down or open surgical removal |
| Other post-discharge complications not listed which is serious, requires further intervention, increases length of stay or causes revisit or hospitalization (adjudicated by principal investigators) |

Supplementary Table 2: Disease Severity

| **Classification of congenital heart disease complexity** |
| --- |
| **MILD:** |
| • Isolated congenital aortic valve disease and bicuspid aortic disease |
| • Isolated congenital mitral valve disease (except parachute valve, cleft leaflet) |
| •Mild isolated pulmonary stenosis (infundibular, valvular, supravalvular) |
| •Isolated small ASD, VSD, or PDA |
| • Repaired secundum ASD, sinus venosus defect, VSD, or PDA without residuae or sequellae, such as chamber enlargement, ventricular dysfunction,or elevated PAP. |
| **MODERATE: (Repaired or unrepaired where not specified; alphabetical order)** |
| Anomalous pulmonary venous connection (partial or total) |
| • Anomalous coronary artery arising from the PA |
| • Anomalous coronary artery arising from the opposite sinus |
| • Aortic stenosis - subvalvular or supravalvular |
| • AVSD, partial or complete, including primum ASD (excluding pulmonary vascular disease) |
| • ASD secundum, moderate or large unrepaired (excluding pulmonary vascular disease) |
| • Coarctation of the aorta |
| • Double chambered right ventricle |
| • Ebstein anomaly |
| • Marfan syndrome and related HTAD, Turner Syndrome |
| • PDA, moderate or large unrepaired (excluding pulmonary vascular disease) |
| • Peripheral pulmonary stenosis |
| • Pulmonary stenosis (infundibular, valvular, supravalvular), moderate or severe |
| • Sinus of Valsalva aneurysm/fistula |
| • Sinus venosus defect |
| • Tetralogy of Fallot -repaired |
| • Transposition of the great arteries after arterial switch operation |
| • VSD with associated abnormalities (excluding pulmonary vascular disease) and/or moderate or greater shunt. |
| **SEVERE: (Repaired or unrepaired where not specified; alphabetical order)** |
| • Any CHD (repaired or unrepaired) associated with pulmonary vascular disease (including Eisenmenger syndrome) |
| • Any cyanotic CHD (unoperated or palliated) |
| • Double-outlet ventricle |
| • Fontan circulation |
| • Interrupted aortic arch |
| • Pulmonary atresia (all forms) |
| • Transposition of the great arteries (except for patients with arterial switch operation) |
| • Univentricular heart (including double inlet left/right ventricle, tricuspid/mitral atresia, hypoplastic left heart syndrome, any other anatomic abnormality with a functionally single ventricle) |
| • Truncus arteriosus |
| • Other complex abnormalities of AV and ventriculoarterial connection (i.e. crisscross heart, heterotaxy syndromes, ventricular inversion). |
| ASD = atrial septal defect; AV = atrioventricular; AVSD = atrioventricular septal defect; CHD = congenital heart disease; HTAD = heritable thoracic aortic disease; LV = left ventricle/ventricular; PA = pulmonary artery; PAP = pulmonary artery pressure; PDA = patent ductus arteriosus; VSD = ventricular septal defect. |

Supplementary Table 3: Procedural Risk Categories

| **Category 1 ( low risk)** |
| --- |
| Atrial septostomy, balloon |
| Atrial septostomy static balloon dilation |
| Balloon angioplasty / native right ventricular outflow tract |
| Balloon angioplasty / proximal pulmonary artery / dilation < 8 atmosphere |
| Balloon angioplasty / right ventricular to pulmonary artery conduit |
| Balloon angioplasty / right ventricular outflow tract s/p surgery (no conduit) |
| Biopsy diagnostic in patient ≥ 10 kg |
| Biopsy post transplant |
| Coil occlusion / device / systemic arterial collaterals |
| Coil occlusion / left superior vena cava |
| Coil occlusion / patent ductus arteriosus |
| Coil occlusion/ Conduit: right atrium to pulmonary artery |
| Coil occlusion / veno-veno collaterals |
| Device closure / atrial septal defect |
| Device closure / fenestration |
| Device closure / patent ductus arteriosus |
| Device closure / patent foramen ovale |
| Device closure / venous collateral |
| Device closure/Conduit: right atrium to pulmonary artery |
| Diagnostic coronary angiogram |
| Coronary angioplasty and stenting |
| Hemodynamic catheterization |
| Interventional techniques / snare foreign body |
| Interventional techniques / trans-septal puncture |
| Invasive procedure / central line placement |
| Invasive procedure / chest tube |
| Invasive procedure / pericardiocentesis elective |
| Other intended hemodynamic alteration / oxygen-nitric trial or inotropes |
| Other procedures: biopsy, bronchoscopy, drains, echo |
| Stent placement / systemic vein |
| Stent redilation / aorta |
| Stent redilation / intracardiac / atria |
| Stent redilation / proximal pulmonary artery |
| Stent redilation / right ventricle to pulmonary artery conduit |
| Stent redilation / systemic artery (not aorta) |
| Stent redilation / systemic vein |
| Intravascular ultrasound |
| Valvuloplasty / pulmonary valve in patient ≥ 1 month of age |
| **Category 2 (Intermediate risk)** |
| Any catheterization within 72 hours of surgery |
| Atrial septostomy dilation and stent / diagnosis not single ventricle |
| Balloon angioplasty / aorta |
| Balloon angioplasty / lobar segment pulmonary artery / dilation < 8 atmosphere and < 4 vessels |
| Balloon angioplasty / proximal pulmonary artery / dilation >= 8 atmosphere |
| Balloon angioplasty / systemic artery (not aorta) |
| Balloon angioplasty / systemic shunt |
| Balloon angioplasty / systemic vein |
| Ballon angioplasty/ Conduit: right or left ventricle or right atrium to pulmonary artery; right atrium to right ventricle; right ventricle to aorta |
| Ballon angioplasty- atrial baffle, Fontan baffle or fenestration (including perforation) |
| Ballon angioplasty- ventricular septal defect |
| Biopsy in patient < 10 kg (not transplant) |
| Coil / coronary fistula |
| Coil occlusion / systemic shunt |
| Coil occlusion/ conduit: right or left ventricle to pulmonary artery |
| Device closure / baffle leak |
| Device closure/ systemic artery |
| Interventional techniques / atherectomy catheter |
| Interventional techniques / recanalization of occluded peripheral vessels |
| Interventional techniques/ recanalization of jailed vessel in stent |
| Invasive procedure / pericardiocentesis emergent |
| Stent placement / aorta |
| Stent placement / intracardiac / atria |
| Stent placement / lobar segment pulmonary artery |
| Stent placement / native right ventricular outflow tract |
| Stent placement / proximal pulmonary artery |
| Stent placement / Right ventricle to pulmonary artery conduit (incl perforation) |
| Stent placement / right ventricular outflow tract after surgery (no conduit) |
| Stent placement / systemic artery (not aorta) |
| Stent redilation / lobar segment pulmonary artery |
| Stent redilation / pulmonary vein |
| Transcatheter placement of valve |
| Valvuloplasty / aorta in patient ≥ 1 month of age |
| Valvuloplasty pulmonary valve in patient < 1 month of age |
| Valvuloplasty tricuspid valve |
| **Category 3 (high risk)** |
| Atrial septostomy dilation and stent |
| Balloon angioplasty / lobar segment pulmonary artery/ dilation ≥ 8 atmosphere and < 4 vessels |
| Balloon angioplasty / lobar segment pulmonary artery and ≥ 4 vessels |
| Balloon angioplasty or stent / pulmonary vein |
| Coil implantation- conduit left ventricle to aorta |
| Device closure / perivalvar leak |
| Device closure / ventricular septal defect |
| Interventional techniques / atretic valve perforation |
| Stent placement / intracardiac / ventricular |
| Stent placement / systemic shunt or ductus arteriosus |
| Stent placement/pulmonary vein |
| Stent placement/ peripheral pulmonary artery |
| Stent redilation / intracardiac / ventricular /ductus arteriosus |
| Valvuloplasty aortic valve in patient < 1 month of age |
| Valvuloplasty mitral valve |
